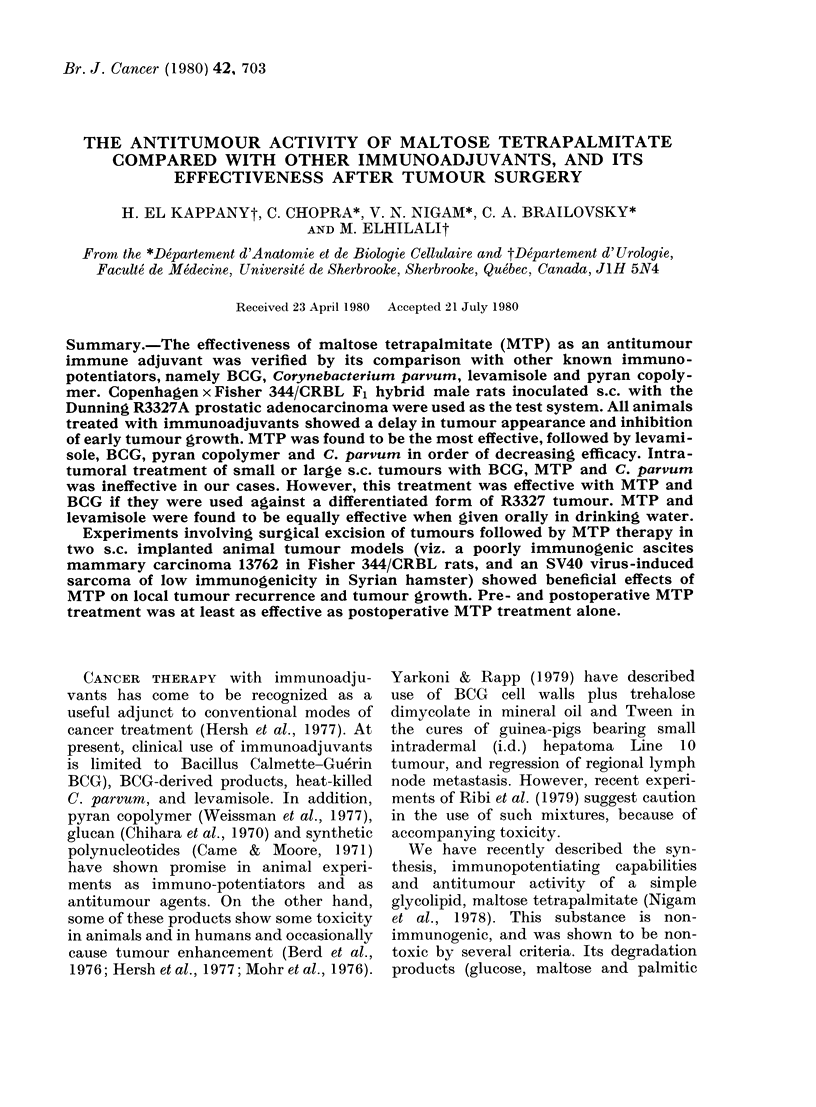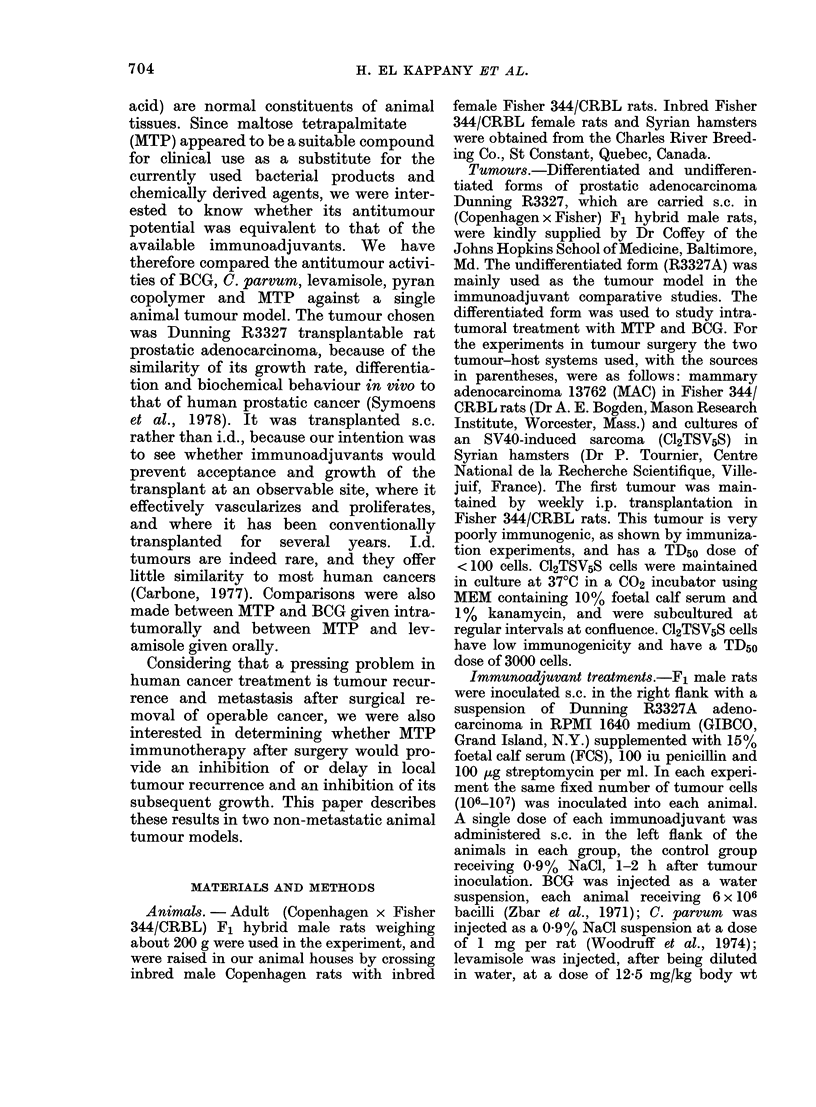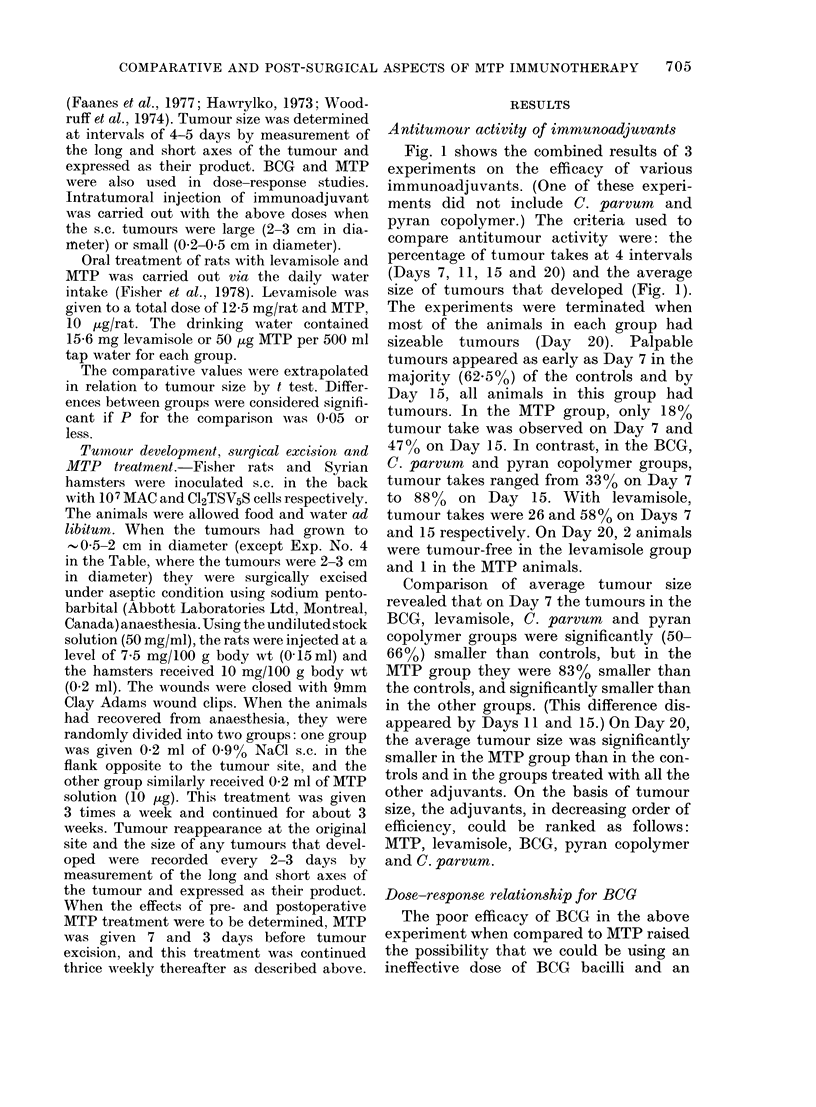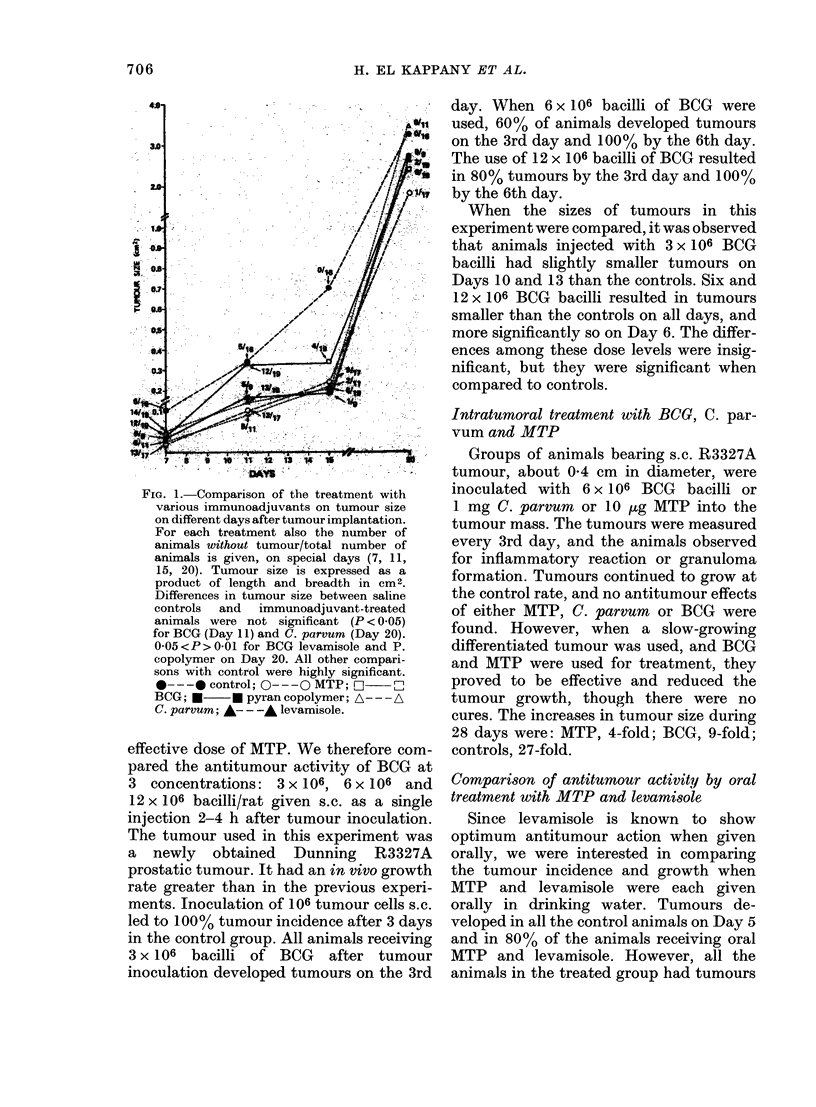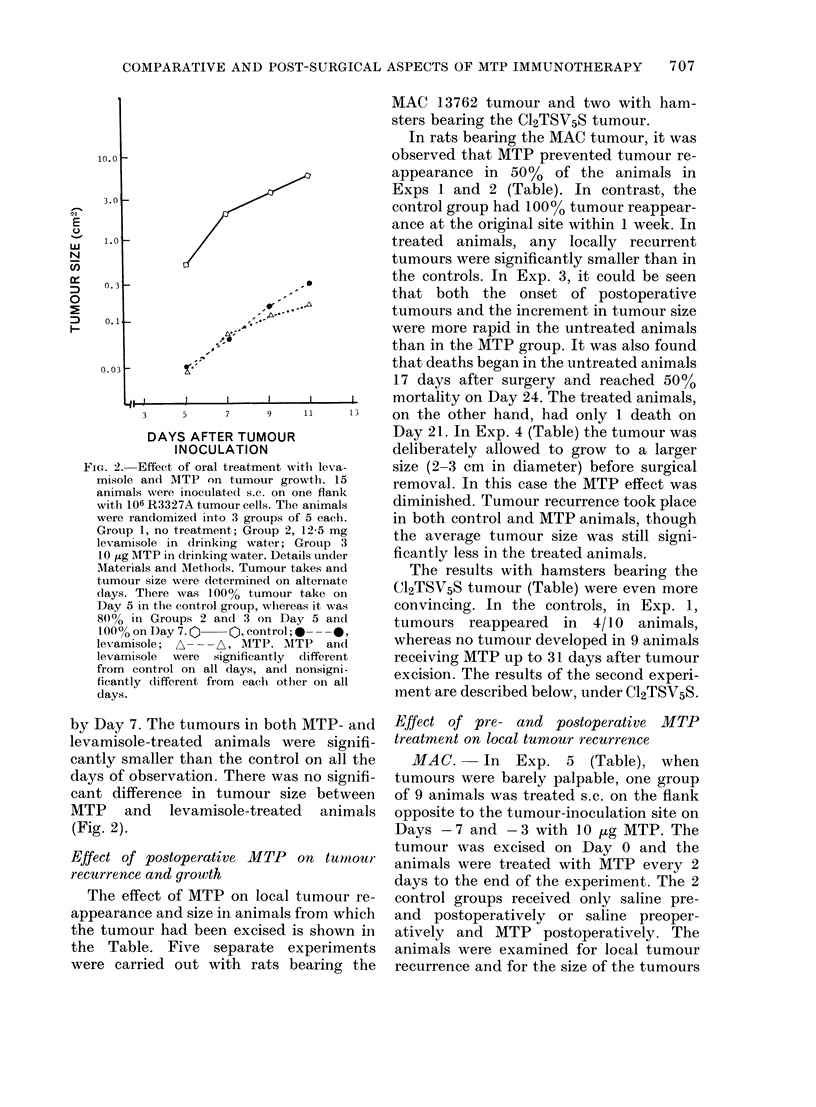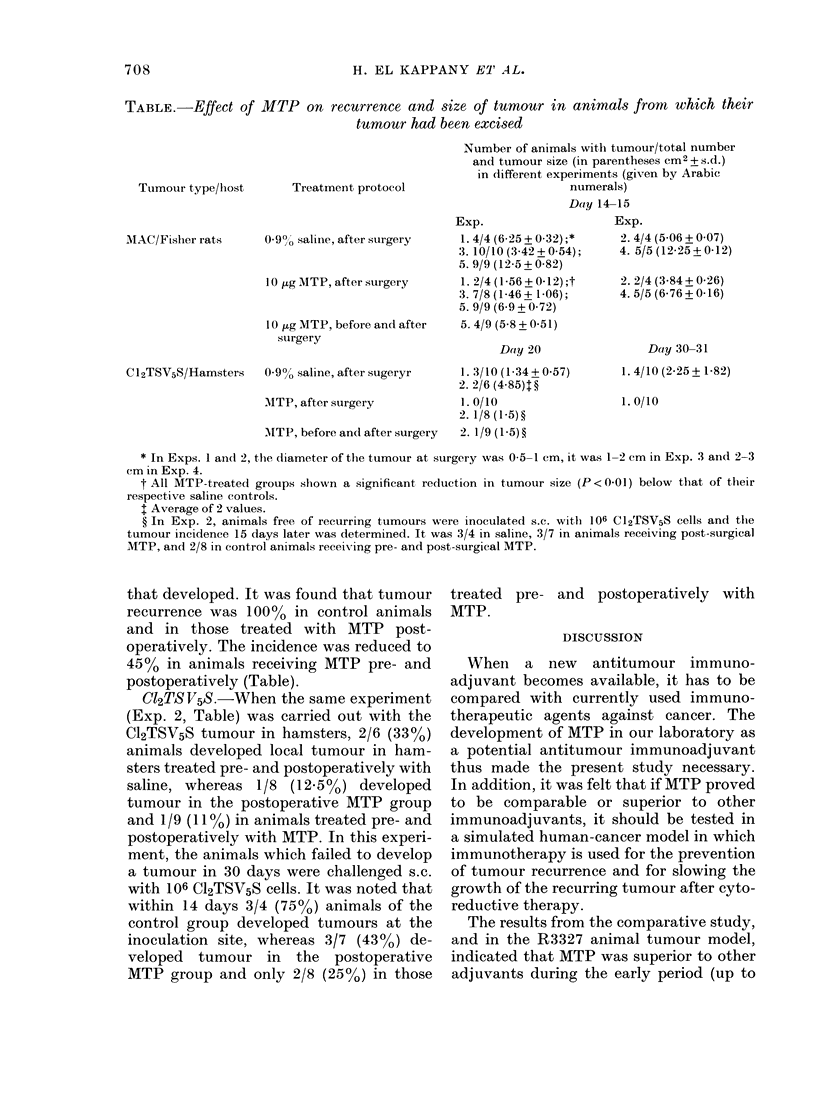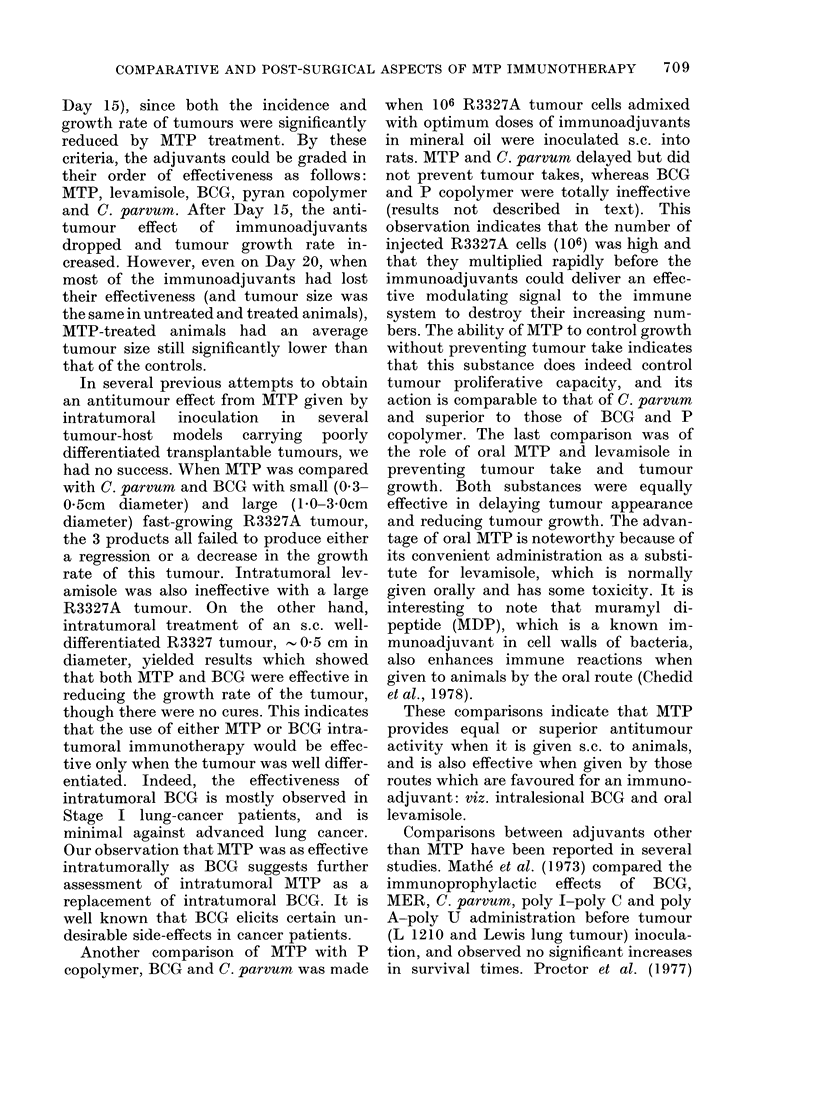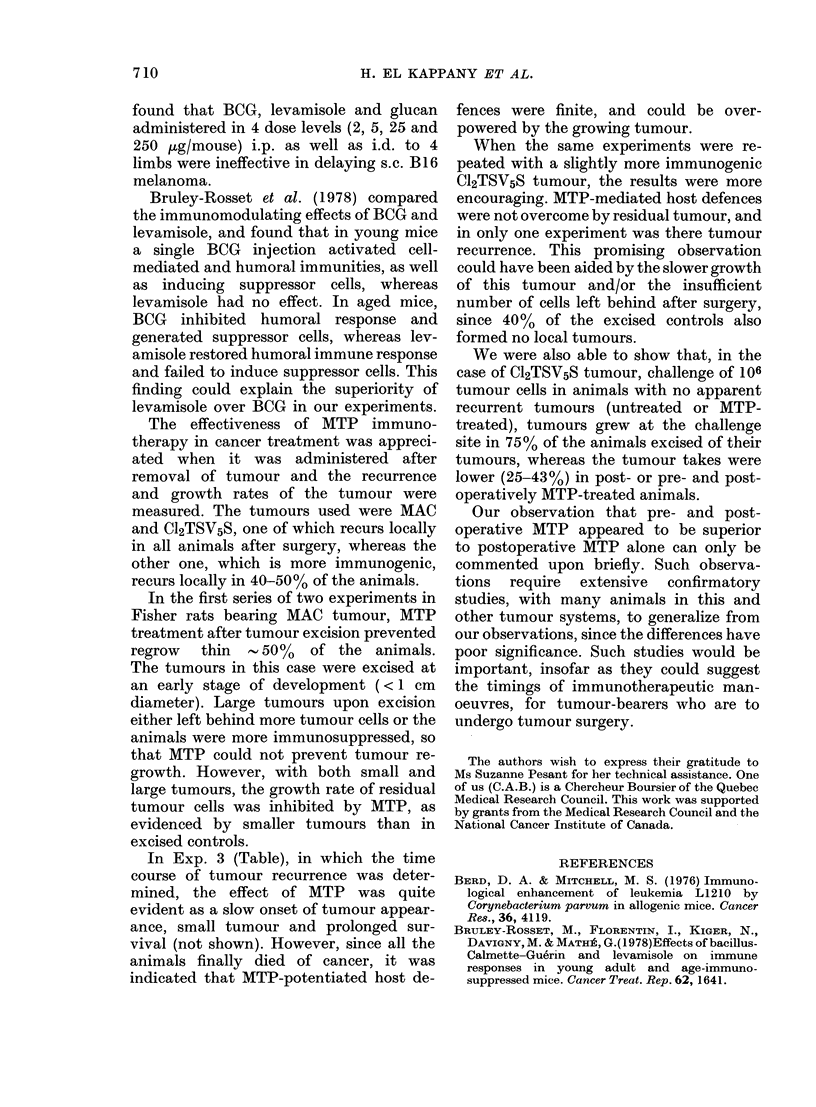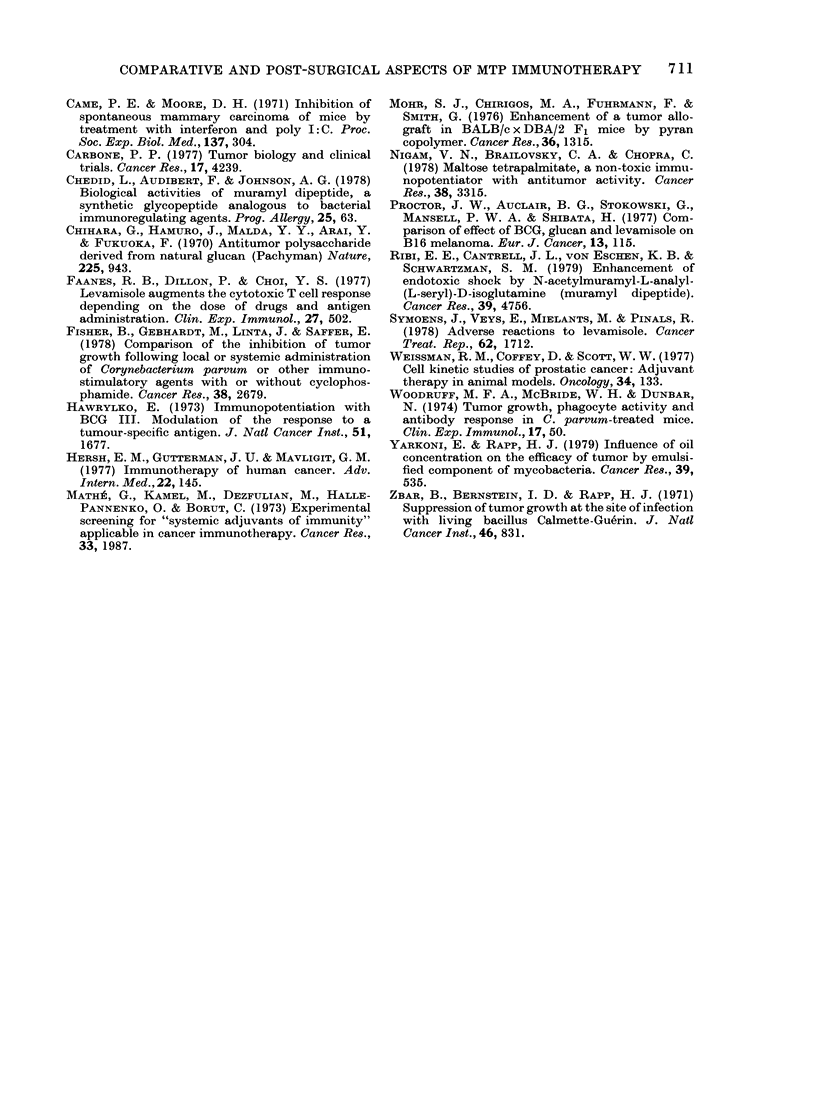# The antitumour activity of maltose tetrapalmitate compared with other immunoadjuvants, and its effectiveness after tumour surgery.

**DOI:** 10.1038/bjc.1980.305

**Published:** 1980-11

**Authors:** H. El Kappany, C. Chopra, V. N. Nigam, C. A. Brailovsky, M. Elhilali

## Abstract

The effectivenss of maltose tetrapalmitate (MTP) as an antitumour immune adjuvant was verified by its comparison with other known immunopotentiators, namely BCG, Corynebacterium parvum, levamisole and pyran copolymer. Copenhagen x Fisher 344/CRBL F1 hybrid male rats inoculated s.c. with the Dunning R3327A prostatic adenocarcinoma were used as the test system. All animals treated with immunoadjuvants showed a delay in tumour appearance and inhibition of early tumour growth. MTP was found to be the most effective, followed by levamisole, BCG, pyran copolymer and C. parvum in order of decreasing efficacy. Intratumoral treatment of small or large s.c. tumours with BCG, MTP and C. parvum was ineffective in our cases. However, this treatment was effective with MTP and BCG if they were used against a differentiated form of R3327 tumour. MTP and levamisole were found to be equally effective when given orally in drinking water. Experiments involving surgical excision of tumours followed by MTP therapy in two s.c. implanted animal tumour models (viz. a poorly immunogenic ascites mammary carcinoma 13762 in Fisher 344/CRBL rats, and an SV40 virus-induced sarcoma of low immunogenicity in Syrian hamster) showed beneficial effects of MTP on local tumour recurrence and tumour growth. Pre- and postoperative MTP treatment was at least as effective as postoperative MTP treatment alone.


*Br. J. Cancer* (1980) **42**, 703

### THE ANTITUMOUR ACTIVITY OF MALTOSE TETRAPALMITATE COMPARED WITH OTHER IMMUNOADJUVANTS, AND ITS EFFECTIVENESS AFTER TUMOUR SURGERY

H. EL KAPPANY†, C. CHOPRA*, V. N. NIGAM*, C. A. BRAILOVSKY* AND M. ELHILALI†

*From the *Département d'Anatomie et de Biologie Cellulaire and †Département d'Urologie, Faculté de Médecine, Université de Sherbrooke, Sherbrooke, Québec, Canada, J1H 5N4*

Received 23 April 1980 Accepted 21 July 1980

**Summary.—The effectiveness of maltose tetrapalmitate (MTP) as an antitumour immune adjuvant was verified by its comparison with other known immunopotentiators, namely BCG, *Corynebacterium parvum*, levamisole and pyran copolymer. Copenhagen × Fisher 344/CRBL $F_1$ hybrid male rats inoculated s.c. with the Dunning R3327A prostatic adenocarcinoma were used as the test system. All animals treated with immunoadjuvants showed a delay in tumour appearance and inhibition of early tumour growth. MTP was found to be the most effective, followed by levamisole, BCG, pyran copolymer and *C. parvum* in order of decreasing efficacy. Intratumoral treatment of small or large s.c. tumours with BCG, MTP and *C. parvum* was ineffective in our cases. However, this treatment was effective with MTP and BCG if they were used against a differentiated form of R3327 tumour. MTP and levamisole were found to be equally effective when given orally in drinking water.**

**Experiments involving surgical excision of tumours followed by MTP therapy in two s.c. implanted animal tumour models (viz. a poorly immunogenic ascites mammary carcinoma 13762 in Fisher 344/CRBL rats, and an SV40 virus-induced sarcoma of low immunogenicity in Syrian hamster) showed beneficial effects of MTP on local tumour recurrence and tumour growth. Pre- and postoperative MTP treatment was at least as effective as postoperative MTP treatment alone.**

CANCER THERAPY with immunoadjuvants has come to be recognized as a useful adjunct to conventional modes of cancer treatment (Hersh *et al.*, 1977). At present, clinical use of immunoadjuvants is limited to Bacillus Calmette–Guérin BCG), BCG-derived products, heat-killed *C. parvum*, and levamisole. In addition, pyran copolymer (Weissman *et al.*, 1977), glucan (Chihara *et al.*, 1970) and synthetic polynucleotides (Came & Moore, 1971) have shown promise in animal experiments as immuno-potentiators and as antitumour agents. On the other hand, some of these products show some toxicity in animals and in humans and occasionally cause tumour enhancement (Berd *et al.*, 1976; Hersh *et al.*, 1977; Mohr *et al.*, 1976). Yarkoni & Rapp (1979) have described use of BCG cell walls plus trehalose dimycolate in mineral oil and Tween in the cures of guinea-pigs bearing small intradermal (i.d.) hepatoma Line 10 tumour, and regression of regional lymph node metastasis. However, recent experiments of Ribi *et al.* (1979) suggest caution in the use of such mixtures, because of accompanying toxicity.

We have recently described the synthesis, immunopotentiating capabilities and antitumour activity of a simple glycolipid, maltose tetrapalmitate (Nigam *et al.*, 1978). This substance is non-immunogenic, and was shown to be non-toxic by several criteria. Its degradation products (glucose, maltose and palmitic acid) are normal constituents of animal tissues. Since maltose tetrapalmitate (MTP) appeared to be a suitable compound for clinical use as a substitute for the currently used bacterial products and chemically derived agents, we were interested to know whether its antitumour potential was equivalent to that of the available immunoadjuvants. We have therefore compared the antitumour activities of BCG, *C. parvum*, levamisole, pyran copolymer and MTP against a single animal tumour model. The tumour chosen was Dunning R3327 transplantable rat prostatic adenocarcinoma, because of the similarity of its growth rate, differentiation and biochemical behaviour *in vivo* to that of human prostatic cancer (Symoens *et al.*, 1978). It was transplanted s.c. rather than i.d., because our intention was to see whether immunoadjuvants would prevent acceptance and growth of the transplant at an observable site, where it effectively vascularizes and proliferates, and where it has been conventionally transplanted for several years. I.d. tumours are indeed rare, and they offer little similarity to most human cancers (Carbone, 1977). Comparisons were also made between MTP and BCG given intratumorally and between MTP and levamisole given orally.

Considering that a pressing problem in human cancer treatment is tumour recurrence and metastasis after surgical removal of operable cancer, we were also interested in determining whether MTP immunotherapy after surgery would provide an inhibition of or delay in local tumour recurrence and an inhibition of its subsequent growth. This paper describes these results in two non-metastatic animal tumour models.

### MATERIALS AND METHODS

*Animals.* — Adult (Copenhagen × Fisher 344/CRBL) $F_1$ hybrid male rats weighing about 200 g were used in the experiment, and were raised in our animal houses by crossing inbred male Copenhagen rats with inbred female Fisher 344/CRBL rats. Inbred Fisher 344/CRBL female rats and Syrian hamsters were obtained from the Charles River Breeding Co., St Constant, Quebec, Canada.

*Tumours.*—Differentiated and undifferentiated forms of prostatic adenocarcinoma Dunning R3327, which are carried s.c. in (Copenhagen × Fisher) $F_1$ hybrid male rats, were kindly supplied by Dr Coffey of the Johns Hopkins School of Medicine, Baltimore, Md. The undifferentiated form (R3327A) was mainly used as the tumour model in the immunoadjuvant comparative studies. The differentiated form was used to study intratumoral treatment with MTP and BCG. For the experiments in tumour surgery the two tumour–host systems used, with the sources in parentheses, were as follows: mammary adenocarcinoma 13762 (MAC) in Fisher 344/CRBL rats (Dr A. E. Bogden, Mason Research Institute, Worcester, Mass.) and cultures of an SV40-induced sarcoma ($Cl_2TSV_5S$) in Syrian hamsters (Dr P. Tournier, Centre National de la Recherche Scientifique, Villejuif, France). The first tumour was maintained by weekly i.p. transplantation in Fisher 344/CRBL rats. This tumour is very poorly immunogenic, as shown by immunization experiments, and has a $TD_{50}$ dose of $<100$ cells. $Cl_2TSV_5S$ cells were maintained in culture at 37°C in a $CO_2$ incubator using MEM containing 10% foetal calf serum and 1% kanamycin, and were subcultured at regular intervals at confluence. $Cl_2TSV_5S$ cells have low immunogenicity and have a $TD_{50}$ dose of 3000 cells.

*Immunoadjuvant treatments.*—$F_1$ male rats were inoculated s.c. in the right flank with a suspension of Dunning R3327A adenocarcinoma in RPMI 1640 medium (GIBCO, Grand Island, N.Y.) supplemented with 15% foetal calf serum (FCS), 100 iu penicillin and 100 $\mu$g streptomycin per ml. In each experiment the same fixed number of tumour cells ($10^6$–$10^7$) was inoculated into each animal. A single dose of each immunoadjuvant was administered s.c. in the left flank of the animals in each group, the control group receiving 0·9% NaCl, 1–2 h after tumour inoculation. BCG was injected as a water suspension, each animal receiving $6 \times 10^6$ bacilli (Zbar *et al.*, 1971); *C. parvum* was injected as a 0·9% NaCl suspension at a dose of 1 mg per rat (Woodruff *et al.*, 1974); levamisole was injected, after being diluted in water, at a dose of 12·5 mg/kg body wt

(Faanes *et al.*, 1977; Hawrylko, 1973; Woodruff *et al.*, 1974). Tumour size was determined at intervals of 4–5 days by measurement of the long and short axes of the tumour and expressed as their product. BCG and MTP were also used in dose–response studies. Intratumoral injection of immunoadjuvant was carried out with the above doses when the s.c. tumours were large (2–3 cm in diameter) or small (0·2–0·5 cm in diameter).

Oral treatment of rats with levamisole and MTP was carried out *via* the daily water intake (Fisher *et al.*, 1978). Levamisole was given to a total dose of 12·5 mg/rat and MTP, 10 μg/rat. The drinking water contained 15·6 mg levamisole or 50 μg MTP per 500 ml tap water for each group.

The comparative values were extrapolated in relation to tumour size by *t* test. Differences between groups were considered significant if *P* for the comparison was 0·05 or less.

*Tumour development, surgical excision and MTP treatment.*—Fisher rats and Syrian hamsters were inoculated s.c. in the back with $10^7$ MAC and $Cl_2TSV_5S$ cells respectively. The animals were allowed food and water *ad libitum.* When the tumours had grown to ~0·5–2 cm in diameter (except Exp. No. 4 in the Table, where the tumours were 2–3 cm in diameter) they were surgically excised under aseptic condition using sodium pentobarbital (Abbott Laboratories Ltd, Montreal, Canada) anaesthesia. Using the undiluted stock solution (50 mg/ml), the rats were injected at a level of 7·5 mg/100 g body wt (0·15 ml) and the hamsters received 10 mg/100 g body wt (0·2 ml). The wounds were closed with 9mm Clay Adams wound clips. When the animals had recovered from anaesthesia, they were randomly divided into two groups: one group was given 0·2 ml of 0·9% NaCl s.c. in the flank opposite to the tumour site, and the other group similarly received 0·2 ml of MTP solution (10 μg). This treatment was given 3 times a week and continued for about 3 weeks. Tumour reappearance at the original site and the size of any tumours that developed were recorded every 2–3 days by measurement of the long and short axes of the tumour and expressed as their product. When the effects of pre- and postoperative MTP treatment were to be determined, MTP was given 7 and 3 days before tumour excision, and this treatment was continued thrice weekly thereafter as described above.

### RESULTS

### *Antitumour activity of immunoadjuvants*

Fig. 1 shows the combined results of 3 experiments on the efficacy of various immunoadjuvants. (One of these experiments did not include *C. parvum* and pyran copolymer.) The criteria used to compare antitumour activity were: the percentage of tumour takes at 4 intervals (Days 7, 11, 15 and 20) and the average size of tumours that developed (Fig. 1). The experiments were terminated when most of the animals in each group had sizeable tumours (Day 20). Palpable tumours appeared as early as Day 7 in the majority (62·5%) of the controls and by Day 15, all animals in this group had tumours. In the MTP group, only 18% tumour take was observed on Day 7 and 47% on Day 15. In contrast, in the BCG, *C. parvum* and pyran copolymer groups, tumour takes ranged from 33% on Day 7 to 88% on Day 15. With levamisole, tumour takes were 26 and 58% on Days 7 and 15 respectively. On Day 20, 2 animals were tumour-free in the levamisole group and 1 in the MTP animals.

Comparison of average tumour size revealed that on Day 7 the tumours in the BCG, levamisole, *C. parvum* and pyran copolymer groups were significantly (50–66%) smaller than controls, but in the MTP group they were 83% smaller than the controls, and significantly smaller than in the other groups. (This difference disappeared by Days 11 and 15.) On Day 20, the average tumour size was significantly smaller in the MTP group than in the controls and in the groups treated with all the other adjuvants. On the basis of tumour size, the adjuvants, in decreasing order of efficiency, could be ranked as follows: MTP, levamisole, BCG, pyran copolymer and *C. parvum.*

### *Dose–response relationship for BCG*

The poor efficacy of BCG in the above experiment when compared to MTP raised the possibility that we could be using an ineffective dose of BCG bacilli and an

FIG. 1.—Comparison of the treatment with various immunoadjuvants on tumour size on different days after tumour implantation. For each treatment also the number of animals *without* tumour/total number of animals is given, on special days (7, 11, 15, 20). Tumour size is expressed as a product of length and breadth in $cm^2$. Differences in tumour size between saline controls and immunoadjuvant-treated animals were not significant ($P < 0{\cdot}05$) for BCG (Day 11) and *C. parvum* (Day 20). $0{\cdot}05 < P > 0{\cdot}01$ for BCG levamisole and P. copolymer on Day 20. All other comparisons with control were highly significant. ●- - -● control; ○- - -○ MTP; □——□ BCG; ■——■ pyran copolymer; △- - -△ *C. parvum*; ▲- - -▲ levamisole.

effective dose of MTP. We therefore compared the antitumour activity of BCG at 3 concentrations: $3 \times 10^6$, $6 \times 10^6$ and $12 \times 10^6$ bacilli/rat given s.c. as a single injection 2–4 h after tumour inoculation. The tumour used in this experiment was a newly obtained Dunning R3327A prostatic tumour. It had an *in vivo* growth rate greater than in the previous experiments. Inoculation of $10^6$ tumour cells s.c. led to 100% tumour incidence after 3 days in the control group. All animals receiving $3 \times 10^6$ bacilli of BCG after tumour inoculation developed tumours on the 3rd day. When $6 \times 10^6$ bacilli of BCG were used, 60% of animals developed tumours on the 3rd day and 100% by the 6th day. The use of $12 \times 10^6$ bacilli of BCG resulted in 80% tumours by the 3rd day and 100% by the 6th day.

When the sizes of tumours in this experiment were compared, it was observed that animals injected with $3 \times 10^6$ BCG bacilli had slightly smaller tumours on Days 10 and 13 than the controls. Six and $12 \times 10^6$ BCG bacilli resulted in tumours smaller than the controls on all days, and more significantly so on Day 6. The differences among these dose levels were insignificant, but they were significant when compared to controls.

### *Intratumoral treatment with BCG,* C. parvum *and MTP*

Groups of animals bearing s.c. R3327A tumour, about 0·4 cm in diameter, were inoculated with $6 \times 10^6$ BCG bacilli or 1 mg *C. parvum* or 10 μg MTP into the tumour mass. The tumours were measured every 3rd day, and the animals observed for inflammatory reaction or granuloma formation. Tumours continued to grow at the control rate, and no antitumour effects of either MTP, *C. parvum* or BCG were found. However, when a slow-growing differentiated tumour was used, and BCG and MTP were used for treatment, they proved to be effective and reduced the tumour growth, though there were no cures. The increases in tumour size during 28 days were: MTP, 4-fold; BCG, 9-fold; controls, 27-fold.

### *Comparison of antitumour activity by oral treatment with MTP and levamisole*

Since levamisole is known to show optimum antitumour action when given orally, we were interested in comparing the tumour incidence and growth when MTP and levamisole were each given orally in drinking water. Tumours developed in all the control animals on Day 5 and in 80% of the animals receiving oral MTP and levamisole. However, all the animals in the treated group had tumours

FIG. 2.—Effect of oral treatment with levamisole and MTP on tumour growth. 15 animals were inoculated s.c. on one flank with $10^6$ R3327A tumour cells. The animals were randomized into 3 groups of 5 each. Group 1, no treatment; Group 2, 12·5 mg levamisole in drinking water; Group 3 10 μg MTP in drinking water. Details under Materials and Methods. Tumour takes and tumour size were determined on alternate days. There was 100% tumour take on Day 5 in the control group, whereas it was 80% in Groups 2 and 3 on Day 5 and 100% on Day 7. ○——○, control; ●– – –●, levamisole; △– – –△, MTP. MTP and levamisole were significantly different from control on all days, and nonsignificantly different from each other on all days.

by Day 7. The tumours in both MTP- and levamisole-treated animals were significantly smaller than the control on all the days of observation. There was no significant difference in tumour size between MTP and levamisole-treated animals (Fig. 2).

*Effect of postoperative MTP on tumour recurrence and growth*

The effect of MTP on local tumour reappearance and size in animals from which the tumour had been excised is shown in the Table. Five separate experiments were carried out with rats bearing the MAC 13762 tumour and two with hamsters bearing the $Cl_2TSV_5S$ tumour.

In rats bearing the MAC tumour, it was observed that MTP prevented tumour reappearance in 50% of the animals in Exps 1 and 2 (Table). In contrast, the control group had 100% tumour reappearance at the original site within 1 week. In treated animals, any locally recurrent tumours were significantly smaller than in the controls. In Exp. 3, it could be seen that both the onset of postoperative tumours and the increment in tumour size were more rapid in the untreated animals than in the MTP group. It was also found that deaths began in the untreated animals 17 days after surgery and reached 50% mortality on Day 24. The treated animals, on the other hand, had only 1 death on Day 21. In Exp. 4 (Table) the tumour was deliberately allowed to grow to a larger size (2–3 cm in diameter) before surgical removal. In this case the MTP effect was diminished. Tumour recurrence took place in both control and MTP animals, though the average tumour size was still significantly less in the treated animals.

The results with hamsters bearing the $Cl_2TSV_5S$ tumour (Table) were even more convincing. In the controls, in Exp. 1, tumours reappeared in 4/10 animals, whereas no tumour developed in 9 animals receiving MTP up to 31 days after tumour excision. The results of the second experiment are described below, under $Cl_2TSV_5S$.

*Effect of pre- and postoperative MTP treatment on local tumour recurrence*

*MAC.* — In Exp. 5 (Table), when tumours were barely palpable, one group of 9 animals was treated s.c. on the flank opposite to the tumour-inoculation site on Days −7 and −3 with 10 μg MTP. The tumour was excised on Day 0 and the animals were treated with MTP every 2 days to the end of the experiment. The 2 control groups received only saline pre- and postoperatively or saline preoperatively and MTP postoperatively. The animals were examined for local tumour recurrence and for the size of the tumours

TABLE.—*Effect of MTP on recurrence and size of tumour in animals from which their tumour had been excised*

| Tumour type/host | Treatment protocol | Number of animals with tumour/total number and tumour size (in parentheses $cm^2 \pm$ s.d.) in different experiments (given by Arabic numerals) | |
|---|---|---|---|
| | | *Day* 14–15 | |
| | | Exp. | Exp. |
| MAC/Fisher rats | 0·9% saline, after surgery | 1. 4/4 (6·25 ± 0·32);* 3. 10/10 (3·42 ± 0·54); 5. 9/9 (12·5 ± 0·82) | 2. 4/4 (5·06 ± 0·07) 4. 5/5 (12·25 ± 0·12) |
| | 10 μg MTP, after surgery | 1. 2/4 (1·56 ± 0·12);† 3. 7/8 (1·46 ± 1·06); 5. 9/9 (6·9 ± 0·72) | 2. 2/4 (3·84 ± 0·26) 4. 5/5 (6·76 ± 0·16) |
| | 10 μg MTP, before and after surgery | 5. 4/9 (5·8 ± 0·51) | |
| | | *Day* 20 | *Day* 30–31 |
| $Cl_2TSV_5S$/Hamsters | 0·9% saline, after sugeryr | 1. 3/10 (1·34 ± 0·57) 2. 2/6 (4·85)‡§ | 1. 4/10 (2·25 ± 1·82) |
| | MTP, after surgery | 1. 0/10 2. 1/8 (1·5)§ | 1. 0/10 |
| | MTP, before and after surgery | 2. 1/9 (1·5)§ | |

* In Exps. 1 and 2, the diameter of the tumour at surgery was 0·5–1 cm, it was 1–2 cm in Exp. 3 and 2–3 cm in Exp. 4.

† All MTP-treated groups shown a significant reduction in tumour size ($P < 0{\cdot}01$) below that of their respective saline controls.

‡ Average of 2 values.

§ In Exp. 2, animals free of recurring tumours were inoculated s.c. with $10^6$ $Cl_2TSV_5S$ cells and the tumour incidence 15 days later was determined. It was 3/4 in saline, 3/7 in animals receiving post-surgical MTP, and 2/8 in control animals receiving pre- and post-surgical MTP.

that developed. It was found that tumour recurrence was 100% in control animals and in those treated with MTP postoperatively. The incidence was reduced to 45% in animals receiving MTP pre- and postoperatively (Table).

*$Cl_2TSV_5S$*.—When the same experiment (Exp. 2, Table) was carried out with the $Cl_2TSV_5S$ tumour in hamsters, 2/6 (33%) animals developed local tumour in hamsters treated pre- and postoperatively with saline, whereas 1/8 (12·5%) developed tumour in the postoperative MTP group and 1/9 (11%) in animals treated pre- and postoperatively with MTP. In this experiment, the animals which failed to develop a tumour in 30 days were challenged s.c. with $10^6$ $Cl_2TSV_5S$ cells. It was noted that within 14 days 3/4 (75%) animals of the control group developed tumours at the inoculation site, whereas 3/7 (43%) developed tumour in the postoperative MTP group and only 2/8 (25%) in those treated pre- and postoperatively with MTP.

### DISCUSSION

When a new antitumour immunoadjuvant becomes available, it has to be compared with currently used immunotherapeutic agents against cancer. The development of MTP in our laboratory as a potential antitumour immunoadjuvant thus made the present study necessary. In addition, it was felt that if MTP proved to be comparable or superior to other immunoadjuvants, it should be tested in a simulated human-cancer model in which immunotherapy is used for the prevention of tumour recurrence and for slowing the growth of the recurring tumour after cytoreductive therapy.

The results from the comparative study, and in the R3327 animal tumour model, indicated that MTP was superior to other adjuvants during the early period (up to

Day 15), since both the incidence and growth rate of tumours were significantly reduced by MTP treatment. By these criteria, the adjuvants could be graded in their order of effectiveness as follows: MTP, levamisole, BCG, pyran copolymer and *C. parvum*. After Day 15, the antitumour effect of immunoadjuvants dropped and tumour growth rate increased. However, even on Day 20, when most of the immunoadjuvants had lost their effectiveness (and tumour size was the same in untreated and treated animals), MTP-treated animals had an average tumour size still significantly lower than that of the controls.

In several previous attempts to obtain an antitumour effect from MTP given by intratumoral inoculation in several tumour-host models carrying poorly differentiated transplantable tumours, we had no success. When MTP was compared with *C. parvum* and BCG with small (0·3–0·5cm diameter) and large (1·0–3·0cm diameter) fast-growing R3327A tumour, the 3 products all failed to produce either a regression or a decrease in the growth rate of this tumour. Intratumoral levamisole was also ineffective with a large R3327A tumour. On the other hand, intratumoral treatment of an s.c. well-differentiated R3327 tumour, ~0·5 cm in diameter, yielded results which showed that both MTP and BCG were effective in reducing the growth rate of the tumour, though there were no cures. This indicates that the use of either MTP or BCG intratumoral immunotherapy would be effective only when the tumour was well differentiated. Indeed, the effectiveness of intratumoral BCG is mostly observed in Stage I lung-cancer patients, and is minimal against advanced lung cancer. Our observation that MTP was as effective intratumorally as BCG suggests further assessment of intratumoral MTP as a replacement of intratumoral BCG. It is well known that BCG elicits certain undesirable side-effects in cancer patients.

Another comparison of MTP with P copolymer, BCG and *C. parvum* was made when $10^6$ R3327A tumour cells admixed with optimum doses of immunoadjuvants in mineral oil were inoculated s.c. into rats. MTP and *C. parvum* delayed but did not prevent tumour takes, whereas BCG and P copolymer were totally ineffective (results not described in text). This observation indicates that the number of injected R3327A cells ($10^6$) was high and that they multiplied rapidly before the immunoadjuvants could deliver an effective modulating signal to the immune system to destroy their increasing numbers. The ability of MTP to control growth without preventing tumour take indicates that this substance does indeed control tumour proliferative capacity, and its action is comparable to that of *C. parvum* and superior to those of BCG and P copolymer. The last comparison was of the role of oral MTP and levamisole in preventing tumour take and tumour growth. Both substances were equally effective in delaying tumour appearance and reducing tumour growth. The advantage of oral MTP is noteworthy because of its convenient administration as a substitute for levamisole, which is normally given orally and has some toxicity. It is interesting to note that muramyl dipeptide (MDP), which is a known immunoadjuvant in cell walls of bacteria, also enhances immune reactions when given to animals by the oral route (Chedid *et al.*, 1978).

These comparisons indicate that MTP provides equal or superior antitumour activity when it is given s.c. to animals, and is also effective when given by those routes which are favoured for an immunoadjuvant: *viz.* intralesional BCG and oral levamisole.

Comparisons between adjuvants other than MTP have been reported in several studies. Mathé *et al.* (1973) compared the immunoprophylactic effects of BCG, MER, *C. parvum*, poly I–poly C and poly A–poly U administration before tumour (L 1210 and Lewis lung tumour) inoculation, and observed no significant increases in survival times. Proctor *et al.* (1977)

found that BCG, levamisole and glucan administered in 4 dose levels (2, 5, 25 and 250 $\mu$g/mouse) i.p. as well as i.d. to 4 limbs were ineffective in delaying s.c. B16 melanoma.

Bruley-Rosset *et al.* (1978) compared the immunomodulating effects of BCG and levamisole, and found that in young mice a single BCG injection activated cell-mediated and humoral immunities, as well as inducing suppressor cells, whereas levamisole had no effect. In aged mice, BCG inhibited humoral response and generated suppressor cells, whereas levamisole restored humoral immune response and failed to induce suppressor cells. This finding could explain the superiority of levamisole over BCG in our experiments.

The effectiveness of MTP immunotherapy in cancer treatment was appreciated when it was administered after removal of tumour and the recurrence and growth rates of the tumour were measured. The tumours used were MAC and $Cl_2TSV_5S$, one of which recurs locally in all animals after surgery, whereas the other one, which is more immunogenic, recurs locally in 40–50% of the animals.

In the first series of two experiments in Fisher rats bearing MAC tumour, MTP treatment after tumour excision prevented regrow thin ~50% of the animals. The tumours in this case were excised at an early stage of development (< 1 cm diameter). Large tumours upon excision either left behind more tumour cells or the animals were more immunosuppressed, so that MTP could not prevent tumour regrowth. However, with both small and large tumours, the growth rate of residual tumour cells was inhibited by MTP, as evidenced by smaller tumours than in excised controls.

In Exp. 3 (Table), in which the time course of tumour recurrence was determined, the effect of MTP was quite evident as a slow onset of tumour appearance, small tumour and prolonged survival (not shown). However, since all the animals finally died of cancer, it was indicated that MTP-potentiated host defences were finite, and could be overpowered by the growing tumour.

When the same experiments were repeated with a slightly more immunogenic $Cl_2TSV_5S$ tumour, the results were more encouraging. MTP-mediated host defences were not overcome by residual tumour, and in only one experiment was there tumour recurrence. This promising observation could have been aided by the slower growth of this tumour and/or the insufficient number of cells left behind after surgery, since 40% of the excised controls also formed no local tumours.

We were also able to show that, in the case of $Cl_2TSV_5S$ tumour, challenge of $10^6$ tumour cells in animals with no apparent recurrent tumours (untreated or MTP-treated), tumours grew at the challenge site in 75% of the animals excised of their tumours, whereas the tumour takes were lower (25–43%) in post- or pre- and post-operatively MTP-treated animals.

Our observation that pre- and post-operative MTP appeared to be superior to postoperative MTP alone can only be commented upon briefly. Such observations require extensive confirmatory studies, with many animals in this and other tumour systems, to generalize from our observations, since the differences have poor significance. Such studies would be important, insofar as they could suggest the timings of immunotherapeutic manoeuvres, for tumour-bearers who are to undergo tumour surgery.

The authors wish to express their gratitude to Ms Suzanne Pesant for her technical assistance. One of us (C.A.B.) is a Chercheur Boursier of the Quebec Medical Research Council. This work was supported by grants from the Medical Research Council and the National Cancer Institute of Canada.

### REFERENCES

Berd, D. A. & Mitchell, M. S. (1976) Immunological enhancement of leukemia L1210 by *Corynebacterium parvum* in allogenic mice. *Cancer Res.*, **36**, 4119.

Bruley-Rosset, M., Florentin, I., Kiger, N., Davigny, M. & Mathé, G. (1978) Effects of bacillus-Calmette–Guérin and levamisole on immune responses in young adult and age-immunosuppressed mice. *Cancer Treat. Rep.* **62**, 1641.

CAME, P. E. & MOORE, D. H. (1971) Inhibition of spontaneous mammary carcinoma of mice by treatment with interferon and poly I:C. *Proc. Soc. Exp. Biol. Med.*, **137**, 304.

CARBONE, P. P. (1977) Tumor biology and clinical trials. *Cancer Res.*, **17**, 4239.

CHEDID, L., AUDIBERT, F. & JOHNSON, A. G. (1978) Biological activities of muramyl dipeptide, a synthetic glycopeptide analogous to bacterial immunoregulating agents. *Prog. Allergy*, **25**, 63.

CHIHARA, G., HAMURO, J., MALDA, Y. Y., ARAI, Y. & FUKUOKA, F. (1970) Antitumor polysaccharide derived from natural glucan (Pachyman) *Nature*, **225**, 943.

FAANES, R. B., DILLON, P. & CHOI, Y. S. (1977) Levamisole augments the cytotoxic T cell response depending on the dose of drugs and antigen administration. *Clin. Exp. Immunol.*, **27**, 502.

FISHER, B., GEBHARDT, M., LINTA, J. & SAFFER, E. (1978) Comparison of the inhibition of tumor growth following local or systemic administration of *Corynebacterium parvum* or other immunostimulatory agents with or without cyclophosphamide. *Cancer Res.*, **38**, 2679.

HAWRYLKO, E. (1973) Immunopotentiation with BCG III. Modulation of the response to a tumour-specific antigen. *J. Natl Cancer Inst.*, **51**, 1677.

HERSH, E. M., GUTTERMAN, J. U. & MAVLIGIT, G. M. (1977) Immunotherapy of human cancer. *Adv. Intern. Med.*, **22**, 145.

MATHÉ, G., KAMEL, M., DEZFULIAN, M., HALLE-PANNENKO, O. & BORUT, C. (1973) Experimental screening for "systemic adjuvants of immunity" applicable in cancer immunotherapy. *Cancer Res.*, **33**, 1987.

MOHR, S. J., CHIRIGOS, M. A., FUHRMANN, F. & SMITH, G. (1976) Enhancement of a tumor allograft in BALB/c x DBA/2 $F_1$ mice by pyran copolymer. *Cancer Res.*, **36**, 1315.

NIGAM, V. N., BRAILOVSKY, C. A. & CHOPRA, C. (1978) Maltose tetrapalmitate, a non-toxic immunopotentiator with antitumor activity. *Cancer Res.*, **38**, 3315.

PROCTOR, J. W., AUCLAIR, B. G., STOKOWSKI, G., MANSELL, P. W. A. & SHIBATA, H. (1977) Comparison of effect of BCG, glucan and levamisole on B16 melanoma. *Eur. J. Cancer*, **13**, 115.

RIBI, E. E., CANTRELL, J. L., VON ESCHEN, K. B. & SCHWARTZMAN, S. M. (1979) Enhancement of endotoxic shock by N-acetylmuramyl-L-analyl-(L-seryl)-D-isoglutamine (muramyl dipeptide). *Cancer Res.*, **39**, 4756.

SYMOENS, J., VEYS, E., MIELANTS, M. & PINALS, R. (1978) Adverse reactions to levamisole. *Cancer Treat. Rep.*, **62**, 1712.

WEISSMAN, R. M., COFFEY, D. & SCOTT, W. W. (1977) Cell kinetic studies of prostatic cancer: Adjuvant therapy in animal models. *Oncology*, **34**, 133.

WOODRUFF, M. F. A., MCBRIDE, W. H. & DUNBAR, N. (1974) Tumor growth, phagocyte activity and antibody response in *C. parvum*-treated mice. *Clin. Exp. Immunol.*, **17**, 50.

YARKONI, E. & RAPP, H. J. (1979) Influence of oil concentration on the efficacy of tumor by emulsified component of mycobacteria. *Cancer Res.*, **39**, 535.

ZBAR, B., BERNSTEIN, I. D. & RAPP, H. J. (1971) Suppression of tumor growth at the site of infection with living bacillus Calmette-Guérin. *J. Natl Cancer Inst.*, **46**, 831.